# Role of genetic variants and DNA methylation of lipid metabolism-related genes in metabolic dysfunction-associated steatotic liver disease

**DOI:** 10.3389/fphys.2025.1562848

**Published:** 2025-03-17

**Authors:** Jun-Jie Wang, Xiao-Yuan Chen, Yi-Rong Zhang, Yan Shen, Meng-Lin Zhu, Jun Zhang, Jun-Jie Zhang

**Affiliations:** ^1^ Key Laboratory of Prevention and Treatment of Cardiovascular and Cerebrovascular Diseases, Ministry of Education, Department of Basic Medicine, Gannan Medical University, Ganzhou, China; ^2^ Department of Publication Health and Health Management, Gannan Medical University, Ganzhou, China

**Keywords:** metabolic dysfunction-associated steatotic liver disease (MASLD), metabolic dysfunction-associated steatohepatitis (MASH), genetic variant, DNA methylation, lipid metabolism, biomarker

## Abstract

Metabolic dysfunction-associated steatotic liver disease (MASLD), is one of the most common chronic liver diseases, which encompasses a spectrum of diseases, from metabolic dysfunction-associated steatotic liver (MASL) to metabolic dysfunction-associated steatohepatitis (MASH), and may ultimately progress to MASH-related cirrhosis and hepatocellular carcinoma (HCC). MASLD is a complex disease that is influenced by genetic and environmental factors. Dysregulation of hepatic lipid metabolism plays a crucial role in the development and progression of MASLD. Therefore, the focus of this review is to discuss the links between the genetic variants and DNA methylation of lipid metabolism-related genes and MASLD pathogenesis. We first summarize the interplay between MASLD and the disturbance of hepatic lipid metabolism. Next, we focus on reviewing the role of hepatic lipid related gene loci in the onset and progression of MASLD. We summarize the existing literature around the single nucleotide polymorphisms (SNPs) associated with MASLD identified by genome-wide association studies (GWAS) and candidate gene analyses. Moreover, based on recent evidence from human and animal studies, we further discussed the regulatory function and associated mechanisms of changes in DNA methylation levels in the occurrence and progression of MASLD, with a particular emphasis on its regulatory role of lipid metabolism-related genes in MASLD and MASH. Furthermore, we review the alterations of hepatic DNA and blood DNA methylation levels associated with lipid metabolism-related genes in MASLD and MASH patients. Finally, we introduce potential value of the genetic variants and DNA methylation profiles of lipid metabolism-related genes in developing novel prognostic biomarkers and therapeutic targets for MASLD, intending to provide references for the future studies of MASLD.

## 1 Introduction

With the rising prevalence of obesity, metabolic syndrome, and type 2 diabetes mellitus (T2DM), metabolic dysfunction-associated steatotic liver disease [MASLD, or previously known as non-alcoholic fatty liver disease (NAFLD)], has emerged as a global epidemic (Younossi et al., 2023). Studies indicate that MASLD affects approximately 30% of the global population, with the highest prevalence observed in Latin America, the Middle East, and North Africa. MASLD comprises a spectrum of disorders, including metabolic dysfunction-associated steatotic liver [MASL, or previously known as non-alcoholic fatty liver (NAFL) ] and metabolic dysfunction-associated steatohepatitis [MASH, or previously known as non-alcoholic steatohepatitis (NASH) ], which can progress to advanced fibrosis, cirrhosis, and liver cancer in certain patients (Loomba et al., 2021). MASH is distinguished from MASL by the presence of hepatic steatosis accompanied by ballooning degeneration of hepatocytes, lobular inflammation, and perisinusoidal fibrosis, marking a critical phase in MASLD progression (Hyun and Jung, 2020). Despite the surge in global incidence of MAFLD and MASH, the complex etiology has hindered the development of effective pharmacological therapies. Consequently, elucidating the molecular mechanisms of MASH and identifying key therapeutic targets are essential for effective prevention and management (Friedman et al., 2018).

Key factors in MASH pathophysiology include hepatocellular lipotoxicity and immune-mediated inflammation (Parthasarathy et al., 2020). Pathological steatosis in MASH results from an imbalance between lipid accumulation, primarily due to fatty acid absorption and *de novo* lipogenesis, and lipid elimination, leading to excessive triglyceride accumulation in hepatic tissue (Jacome-Sosa and Parks, 2014). Notably, variations in MASLD incidence and severity among individuals, along with disparities among ethnic groups, underscore the importance of genetic and epigenetic contributions to the disease’s development and progression. Over the past 15 years, genome-wide association studies (GWAS) have identified several genetic loci associated with MASLD and/or MASH (Anstee and Day, 2013; Anstee et al., 2020; Vujkovic et al., 2022). A recent GWAS meta-analysis reported 17 novel variants linked to MASLD (Chen et al., 2023b), enhancing our understanding of MASLD etiology (Sharma and Mandal, 2022). Alongside genetic factors, the impact of epigenetics on metabolic diseases has garnered increasing attention. Environmental factors, such as nutrition, smoking, and air pollution, induce epigenetic modifications that significantly contribute to MASLD and MASH progression (Ramezani et al., 2023). These alterations affect gene expression, influencing phenotypic outcomes. Numerous studies have linked epigenetic changes—such as DNA methylation patterns, microRNA (miRNA) expression, and histone modifications—to MASLD’s onset and progression. Establishing an epigenetic profile indicative of disease status could enhance individual risk assessments for MASLD. Importantly, epigenetic modifications are heritable and reversible, offering potential avenues for innovative personalized prevention and treatment strategies. DNA methylation, one of the most studied epigenetic mechanisms, serves as a paradigm of epigenetic regulation. The field has seen a consistent increase in discoveries driven by ongoing research and technological advancements (Mattei et al., 2022).

Lipid metabolism is fundamental to various diseases linked to inflammation and metabolic dysfunction. Numerous single nucleotide polymorphisms (SNPs) and methylation-modifiable loci exist within key genes involved in lipid metabolism (Jonas and Schürmann, 2021). These genetic variants and epigenetic modifications significantly influence MASLD’s development and progression. Therefore, understanding the genetic and epigenetic factors within lipid metabolism-related genes is critical for addressing the rising incidence of MASLD. This review focuses on the genetic and epigenetic changes in lipid metabolism-related genes associated with MASLD risk, providing insights into their potential applications for risk assessment.

## 2 Disturbance of hepatic lipid metabolism in MASLD and MASH

**FIGURE 1:**
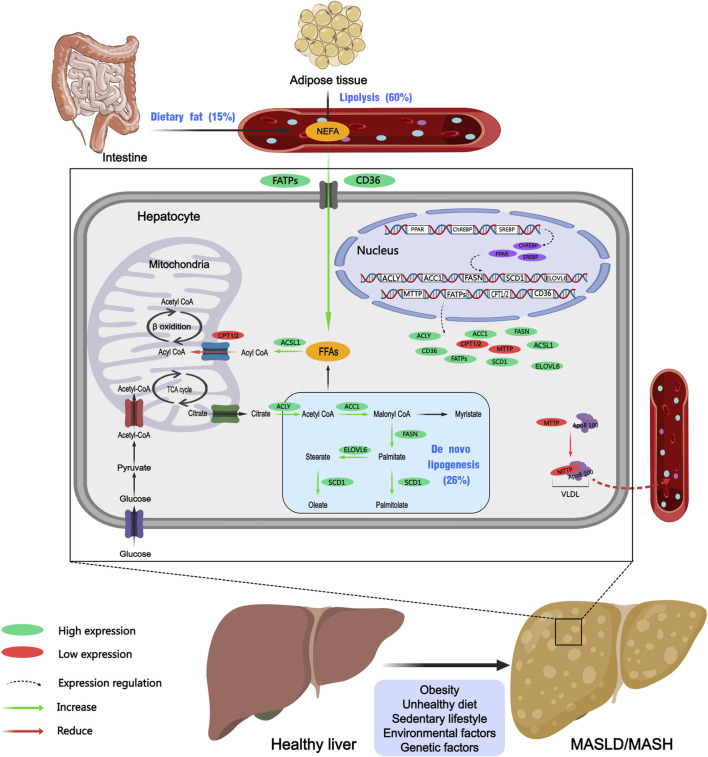
Dysregulation of hepatic lipid metabolism in MASLD and MASH. Purple ovals represent transcription factors, green ovals indicate upregulated proteins, red oals denote downregulated proteins, dashed arrows signify regulatory processes, green arrows represent promoting reactions, and red arrows indicate inhibitory reactions. Created with MedPeer (medpeer.cn). TCA cycle, tricarboxylic acid cycle; ACC1, acetyl-CoA carboxylase 1; FASN, fatty acid synthase; SCD1, stearoyl-CoA desaturase 1; FATPs, fatty acid transport Proteins; CD36, cluster of differentiation 36; ACSL1, acyl-CoA synthetase long-chain family member 1; CPT1/2, carnitine palmitoyltransferase 1/2; MTTP, microsomal triglyceride transfer protein; ApoB-100, apolipoprotein B-100; ChREBP, carbohydrate response element binding protein; PPAR, peroxisome proliferator-activated receptor; SREBP, sterol regulatory element-binding protein; NEFA, non-esterified fatty acids; FFAs, free fatty acids.

Dyslipidemia, along with insulin resistance, metabolic syndrome (MetS), obesity, and T2DM, are major risk factors for MASLD and play key roles in the onset and progression of MASLD (Powell et al., 2021). Given this context, macrophage infiltration in visceral adipose tissue triggers a pro-inflammatory state that exacerbates insulin resistance. Simultaneously, increased lipolysis results in a substantial influx of free fatty acids into the liver, which, alongside an elevation in *de novo* lipogenesis (DNL) and reduction in liver metabolic capacity. The lipid metabolism disorder contributes to the accumulation of hepatotoxic lipids, which are related to a worse histological profile in MASLD. In patients with MASLD, the accumulation of lipids, including triglycerides, cholesteryl esters, and phospholipids, particularly triglycerides, leads to the formation of intracellular lipid droplets in hepatocytes (Musso et al., 2013b; Jacome-Sosa and Parks, 2014). Research indicates that approximately 60% of hepatic triglycerides originate from free fatty acids released by adipose tissue, 26% are synthesized through *de novo* lipogenesis (DNL), and 15% are derived from dietary sources (Figure 1) (Donnelly et al., 2005). The accumulation of lipids in the liver is associated with an imbalance in hepatic lipid metabolism, involving the uptake and export of fatty acids, *de novo* lipogenesis, and fatty acid utilization (Badmus et al., 2022). Prolonged accumulation can activate inflammatory and fibrotic processes, thereby exacerbating liver disease.

Abnormalities in lipid uptake by the liver are significant contributors to the development of MASLD. Fatty acid transport proteins (FATPs) and cluster of differentiation 36 (CD36) play crucial roles in regulating the hepatic uptake of lipids. Alterations in the promoter region of the FATP5 gene are associated with increased FATP5 expression, which exacerbates steatosis in the liver of MASLD patients (Auinger et al., 2010). CD36 is a glycoprotein receptor located on the cell membrane that functions as a fatty acid transporter. It facilitates the delivery of long-chain fatty acids into cells, thereby playing a significant role in energy metabolism. Study demonstrated that enhanced CD36 expression in the liver of patients with MASLD correlated with increased hepatic fat content (Miquilena-Colina et al., 2011). Additionally, elevated levels of CD36 gene expression have been observed in the liver tissue of mice subjected to a high-fat diet (HFD) (Buttet et al., 2016).

Abnormal DNL is another key factor contributing to the development and progression of MASLD and MASH. Enzymes such as acetyl-CoA carboxylase (ACC), fatty acid synthase (FASN), and stearoyl-CoA desaturase-1 (SCD1) are involved in regulating DNL in the liver. Studies revealed that transcription factors like peroxisome proliferator-activated receptor gamma (PPARγ), sterol regulatory element-binding protein-1 (SREBP-1), and carbohydrate-responsive element-binding protein (ChREBP) increase the expression and activity of multiple enzymes, including FASN, ACC and SCD1 (Buzzetti et al., 2016; Badmus et al., 2022). ChREBP is predominantly expressed in hepatic tissue, where it regulates the synthesis, elongation, and degradation of fatty acids by activating key enzymes such as ACC1, FASN, ELOVL6, and SCD1 (Iizuka et al., 2020). In another study, Dentin et al. (2006) investigated the effects of hepatic ChREBP inhibition in ob/ob mice, and discovered that it significantly improved hepatic steatosis by selectively suppressing lipogenesis, this intervention resulted in a reduction in plasma triglyceride and non-esterified fatty acid (NEFA) content. Moreover, the sterol regulatory element-binding proteins (SREBPs) are encoded by the genes SREBF1 and SREBF2. Specifically, SREBP-1c regulates numerous genes associated with lipid metabolism, including ACC and FASN, which are two key enzymes involved in DNL (Rong et al., 2017; Shimano and Sato, 2017). Similar experimental studies have implied that in 3 mouse models like ob/ob mice, high-fat diet (HFD)-induced mice, and sucrose diet-induced mice, the absence of SREBPs can reduce lipid synthesis, ameliorate pathological lipid accumulation, and prevent the development of MASLD (Moon et al., 2012). Peroxisome proliferator-activated receptors (PPARs) exist in three distinct isoforms: PPARα, PPARδ, and PPARγ (Chen et al., 2023a). These PPARs are transcription factors belonging to the nuclear receptor superfamily, which play essential roles in MASLD by modulating key biological processes such as inflammation, lipid and glucose metabolism, and energy homeostasis. Peroxisome proliferator-activated receptor alpha (PPARα) is highly expressed in hepatocytes and plays a critical role in the regulation of lipid transport and metabolism (Kersten, 2014). There is evidence that the ablation of PPARα in hepatocytes induces hepatic steatosis and inflammation in mice fed a HFD (Regnier et al., 2020). Peroxisome proliferator-activated receptor gamma (PPARγ) is the most extensively studied isoform, primarily responsible for regulating adipocyte differentiation, lipogenesis, and various metabolic functions (Chen et al., 2023a). PPARγ stimulates the hepatic production of FASN, resulting in elevated triglyceride levels within hepatocytes. Moreover, it enhances the transcription of sterol regulatory element-binding protein 1c (SREBP1c), which activates additional lipogenic genes and accelerates the conversion of pyruvate into fatty acids. In 2011, Moran-Salvador et al. (2011) have found that hepatocyte-specific knockdown of PPARγ in murine models led to a reduction in hepatic steatosis. This finding indicated that PPARγ was a major regulator of hepatic lipogenesis and significantly influenced lipid accumulation in the liver.

Apart from the regulation of DNL, triglyceride secretion constitutes another vital mechanism for controlling hepatic lipid concentrations. Essential components in this process include apolipoprotein B100 (ApoB100) and MTTP. Within the endoplasmic reticulum (ER), MTTP facilitates the lipidation of ApoB100, leading to the formation of VLDL particles (Hinds et al., 2020). Genetic abnormalities in MTTP can impair hepatic triglyceride secretion, causing the occurrence of MASLD. A separate study investigating the susceptibility of the Han Chinese population to MASLD identified that genetic polymorphisms in the MTTP gene may influence the risk of developing MASLD (Peng et al., 2014).

Over all, the progression of MASLD and MASH is intricately linked to hepatic fatty acid accumulation, triglyceride synthesis, and the expression of various regulatory enzymes. Genetic variations and differential expression of these metabolic pathways may either elevate or lower the risk of MASLD and MASH. A comprehensive understanding of these fundamental processes would facilitate the development of more efficacious therapeutic strategies for the prevention and treatment of these increasingly prevalent hepatic disorders.

## 3 Hepatic lipid related gene loci associated with MASLD and MASH susceptibility

**FIGURE 2:**
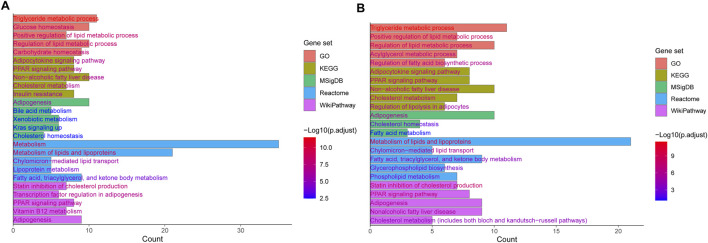
Top 5 enriched terms and pathways of genes **(A)** and lipid-related genes **(B)** affected by MASLD genetic risk variants determined by GO, KEGG, MSigDB, Reactome, and WikiPathways.

Genetic factors play a critical role in the onset and progression of MASLD and MASH. Previous studies have identified a large amount of genetic variants associated with MASLD (Del Campo et al., 2018; Lin et al., 2020). These variants take part in numerous pathogenic pathways, including lipid metabolism, insulin signaling, oxidative stress, inflammation, and fibrosis, which contribute to the significant variability in the incidence and mortality among individuals with MASLD-related disorders. MASLD and MASH exhibit a strong heritable component estimated to range from 35% to 61% (Loomba et al., 2021). Nevertheless, the genetic variants identified thus far account for only a minor fraction (10%–20%) of the total heritability (Eslam and George, 2019). Indeed, MASLD is a polygenic disorder, and developing predictive models requires more comprehensive genetic information. To date, numerous studies have detected a variety of genetic variants associated with MASLD and MASH through genome-wide association studies (GWAS) (Donati et al., 2016; Abul-Husn et al., 2018; Chung et al., 2018; Riccio et al., 2022; Xia et al., 2022). In 2008, Romeo et al. (2008) conducted the first GWAS for MASLD, and identified a SNP (rs738409 C>G) in PNPLA3 significantly associated with MASLD. Since then, more and more groups discovered SNPs linked to lipid metabolism-related genes in patients with MASLD using GWAS or candidate gene analyses (Chung et al., 2018; Kawaguchi et al., 2018; Anstee et al., 2020; Chen et al., 2023b). Those studies identified a number of candidate genes including PNPLA3, GCKR, TM6SF2, HSD17B13, MBOAT7, PPP1R3B, SAMM50, NCAN, LYPLAL1 and FDFT1 (Table 1). The associations between these genetic variants and elevated levels of serum alanine aminotransferase (ALT) and aspartate aminotransferase (AST) have been confirmed. Furthermore, these variants are also linked to hepatic conditions such as simple steatosis, steatohepatitis, liver cirrhosis, and HCC. Additionally, they are frequently associated with metabolic disorders including type 2 diabetes, obesity, and cardiovascular diseases (Sharma and Mandal, 2022). Notably, in a recent study, Upadhyay et al. (2023) conducted a GWAS based on three large databases related to hepatic steatosis, consisting of the UK Biobank (UKBB), the Michigan Genomics Initiative (MGI), and the Genetics of Obesity-related Liver Disease (GLOD) Consortium. They ultimately identified a significant variant rs6461378 (C>T; SUN1 p.H118Y) that showed closely association with fatty degeneration. This association was further confirmed across multiple ethnically diverse validation cohorts and various cell lines, linking rs6461378 to histological MASLD and MASLD-associated metabolic characteristics. Their findings suggested that SUN1 may serve as a promising and relevant therapeutic target for MASLD/MASH and metabolic diseases. In this study, we summarized the existing literature around the SNPs associated with MASLD and MASH identified by GWAS and candidate gene analyses, and obtained 138 SNPs in 98 genes (Supplementary Table S1) (Iwamoto et al., 2001; Nozaki et al., 2004; Sazci et al., 2008; Carulli et al., 2009; Kotronen et al., 2009; Musso et al., 2009; Yan et al., 2009; Yoneda et al., 2009; Chalasani et al., 2010; Dongiovanni et al., 2010; Oliveira et al., 2010; Teslovich et al., 2010; Valenti et al., 2010; Swellam and Hamdy, 2012; Valenti et al., 2012; Adams et al., 2013; Musso et al., 2013a; Vazquez-Chantada et al., 2013; Xu, 2013; Di Filippo et al., 2014; Fares et al., 2014; Li, 2014; Nobili et al., 2014; Wang et al., 2014a; Wang et al., 2014b; Lutz et al., 2016; Tan et al., 2016; Wallace et al., 2016; Nakajima et al., 2017; Hudert et al., 2018; Kovalic et al., 2018; Dongiovanni et al., 2019; Esteve-Luque et al., 2021; Wang et al., 2021; Buzova et al., 2022; Luukkonen et al., 2023a; Lee et al., 2024). Enrichment analyses were performed using EnrichR/gseapy combined with different databases (GO_Biological_Process_2021_Human, KEGG_2021_Human, MSigDB_Hallmark_2020_Human, Reactome_2016_Human and WikiPathway_2021_Human) to determine the potential functions of 98 candidate genes in the pathogenesis of MASLD (Supplementary Table S2; Figure 2A). We observed that these genes were predominantly associated with multiple lipid and glucose metabolism-related pathways. Subsequently, we screened lipid-related pathways using the keywords such as lipid, fatty acid, fat, cholesterol and steroid, and identified 59 unique genes enriched in lipid biosynthetic process, fatty acid metabolic process, cholesterol homeostasis and cholesterol biosynthesis pathway, PPAR signaling pathway and MASLD (Supplementary Tables S3, S4; Figure 2B). The gene list encompass genes identified in multiple studies across diverse populations that were closely associated with MASLD and MASH, including PNPLA3, TM6SF2, HSD17B13, GCKR, members of the FADS family, FDFT1, and PPP1R3B.

**TABLE 1:** List of main genetic variants of lipid metabolism-related genes associated with the development of MASLD and/or progression to MASH and fibrosis and relative clinical phenotype.

Chr	Variant	Gene	Consequence	Function	Reference allele	Alternate allele	Population (ethnicity)	Subjects (n)	Phenotype	Reference
22	rs738409	PNPLA3	p.I148M	Lipid droplets remodeling	C	G	Hispanic	383	↑ MASLD, MASH, hepatic fat content, ALT	Romeo et al. (2008)
							African American	1,032		
							European American	696		
							Chinese	5,581		Xia et al. (2022)
							Korean	2,337		Chung et al. (2018)
	rs6006460		p.S543I		G	T	African American	1,032	↓ Hepatic fat content	Romeo et al. (2008)
	rs12483959		Intron variant		G	A	Korean	2,337	↑ MASLD, ALT	Chung et al. (2018)
	rs2281135		Intron variant		G	A	Korean	2,337	↑ MASLD, ALT	
	rs2294918		p.K434E		A	G	Italian	142	↓ p.I148M	Donati et al. (2016)
19	rs58542926	TM6SF2	p.G167L	VLDL secretion	C	T	Chinese	5,581	↑ MASLD, MASH, liver fat content	Xia et al. (2022)
							Finnish	10		Boren et al. (2020)
4	rs72613567	HSD17B13	Splice donor variant	Lipid remodeling	A	AA	European	46,544	↓ MASLD, ALT, AST, I148 M	Abul-Husn et al. (2018)
							Danish	111,612		Gellert-Kristensen et al. (2020a)
							Spanish/Latin	9,342		Kallwitz et al. (2020)
							Asian	165		Ting et al. (2021)
	rs62305723		p.P260S		G	A	Asian	165	↓ MASLD, MASH	
2	rs780094	GCKR	Intron variant	*De novo* lipogenesis regulation	T	C	Chinese	733	↑ MASLD, hepatic fat content, TG	Yuan et al. (2022)
	rs1260326		p.L446P		T	C	Chinese	733	↑ MASLD, hepatic fat content, TG	
							Japanese	902		Kawaguchi et al. (2018)
19	rs641738	MBOAT7	p.G17V	Remodeling of phosphatidylinositol	T	C	Chinese	5,581	↑ MASLD, Fibrosis	Xia et al. (2022)
							European	1,149		Mancina et al. (2016)
22	rs2143571	SAMM50	Intron variant	Control of reactive oxygen species, mitochondrial shape, cell division	G	A	Korean	2,337	↑ MASLD, ALT	Chung et al. (2018)
	rs3761472		p.Asp110Gly		A	G	Korean	2,337		
	rs2073080				C	T	Korean	2,337		
	rs738491		Intron variant		C	T	Han Chinese	380	↑ MASLD, triglycerides, ALT, AST	Li et al. (2021c)
	rs2073082		Intron variant		G	A	Han Chinese	380		
11	rs174537	FADS1	Intron variant	Fatty acid metabolism	G	T	African American European American	329	↑ AA	Mathias et al. (2011)
11	rs174616	FADS2	Intron variant	Fatty acid metabolism	G	A	Finnish	95	↑ MASLD	Walle et al. (2019)
19	rs2228603	NCAN	p.P92S	Nervous system signaling	C	T	European	7,176	↑ MASL	McCarthy et al. (2011)
8	rs4240624	PPP1R3B	Intron variant	Glycogen synthesis	G	A	European	7,176	↑ MASL	
1	rs12137855	LYPLAL1	Intron variant	TGL catabolism	C	T	European	7,176	↑ MASLD	
8	rs2645424	FDFT1	Intron variant	Modulate intrahepatic cholesterol biosynthesis	A	G	Caucasians African Americans Hispanics	229	↑ MASLD	Santoro et al. (2013)
7	rs6461378	SUN1	p.H118Y	Nuclear envelope structure, cell nucleus, cytoskeleton	C	T	Multiethnic	120,000	Simple steatosis, MASLD	Upadhyay et al. (2023)

The PNPLA3 gene encodes a triglyceride lipase, which plays a critical role in lipid metabolism, and many studies have suggested that PNPLA3 is closely related to the pathogenesis of MASLD. In the beginning, research on the role of PNPLA3 SNPs in risk for MASLD has been centered on rs738409 (Romeo et al., 2008). This SNP, encodes the PNPLA3 I148M variant, results in an amino acid change from isoleucine to methionine, which has been recognized as the most commonly associated variants for MASLD in Hispanic, African American, and European American populations, and has strongly correlation with increased liver fat and hepatic inflammation. Additionally, the study identified another variant in the PNPLA3 gene (rs6006460), involving a thymine-to-cytosine nucleotide alteration that is associated with reduced hepatic fat content in African Americans. The associations between PNPLA3 genetic variants and MASLD were further validated by a large number of subsequent GWAS using populations from Asia, Europe, and the Americas (Lin et al., 2014; Shang et al., 2015; Anstee et al., 2020; Gellert-Kristensen et al., 2020b; Xia et al., 2022). In 2022, a comprehensive genomic analysis identified strong associations between PNPLA3 (rs738409), MBOAT7 (rs641738), and TM6SF2 (rs58542926) and increased hepatic fat accumulation (Xia et al., 2022). Researchers calculated a genetic susceptibility score for MASLD by aggregating these risk alleles, and they discovered a positive correlation between the genetic susceptibility score and the liver fat content (LFC) as well as serum levels of ALT. Additionally, the genetic score exhibited an inverse correlation with plasma triglyceride and cholesterol levels. Individuals with elevated genetic susceptibility scores demonstrated markedly increased rates of liver-specific mortality. Moreover, Chung et al. (2018) performed a GWAS on a Korean cohort to investigate SNPs associated with MASLD. The discovery cohort consisted of 1,593 individuals with MASLD and 2,816 healthy controls, while the validation cohort included 744 individuals with MASLD and 1,137 healthy controls. In both cohorts, significant associations were identified between SNPs in the PNPLA3 (rs738409, rs12483959, and rs2281135) and MASLD. Associations were also observed for SNPs in the SAMM50 (rs2143571, rs3761472, and rs2073080). These six SNPs were significantly correlated with the severity of hepatic steatosis, the incidence of MASLD, and elevated serum alanine aminotransferase (ALT) levels. Although the significance of PNPLA3 in MASLD has been repeatedly validated through GWAS, the underlying pathogenic mechanisms remain incompletely understood. Notably, in a recently study by Luukkonen et al. (2023b), which employed advanced stable isotope techniques to investigate the hepatic metabolic effects in individuals carrying the GG risk homozygosis for the PNPLA3 I148M variant. Their findings revealed an intrinsic mechanism characterized by impaired hepatic mitochondrial function, evidenced by significantly increased ketogenesis alongside reduced lipogenesis and decreased mitochondrial citrate synthase flux. This study helps us understand why homozygous carriers of the PNPLA3 I148M variant were at an elevated risk for progressive liver disease. Meanwhile, other variants of PNPLA3 warrant our attention. Donati et al. (2016) discovered that the PNPLA3 variant rs2294918 (A>G), which encodes the PNPLA3 K434E substitution, reduces PNPLA3 expression and mitigates the impact of the I148M variant on susceptibility to hepatic steatosis and liver injury. Although the K434E variant does not exhibit as strong predictive power as the I148M variant, it remains highly important for elucidating how genetic risk factors of PNPLA3 influence the development of MASLD.

TM6SF2 is a transmembrane protein predominantly expressed in the liver, kidneys, and small intestine. TM6SF2 plays a crucial role in hepatic lipid metabolism, affecting the secretion of triglycerides and the composition of lipid droplets within the liver. Dysfunction of TM6SF2 has been associated with the onset of MASLD (Kozlitina et al., 2014; Mahdessian et al., 2014). The TM6SF2 variant rs58542926 (C>T), which results in an amino acid substitution at position 167, from glutamate to lysine (E167K), has been identified as one of the significant genetic determinants of hepatic fat content (Romeo et al., 2020). Researchers in Finland applied isotope tracing methods to explore the association between the TM6SF2 E167K variant and reduced levels of MASLD and plasma triglycerides in humans Boren et al. (2020), which involved in 10 homozygote carriers for TM6SF2 E167K and 10 controls. Their findings demonstrated that the TM6SF2 E167K variant was directly linked to a decrease in the hepatic production of very low-density lipoprotein 1 (VLDL1). According to the proposed mechanism, the body’s ability to secrete large, triglyceride-rich lipoproteins is compromised, resulting in an inefficient VLDL pathway, which leads to lipid accumulation in the liver. In a recent study, Chen et al. (2024) systematically estimated the effects on hepatic steatosis of dietary patterns alongside genetic variants including PNPLA3-rs738409-G, TM6SF2-rs58542926-T, a polygenic risk score (PRS) based on 16 variants, and their interactions among 21,619 participants from the UK Biobank (UKBB). An association analysis was conducted between those factors and LFC, which indicated that these genetic factors exacerbated the impact of diet on hepatic steatosis, inflammation, and fibrosis.

The 17β-hydroxysteroid dehydrogenase (HSD17B) enzyme family consists of 15 isoenzymes that play important roles in various metabolic processes (Su et al., 2019). These enzymes are essential for the metabolism of steroid hormones, cholesterol, fatty acids, and bile acids, consequently, they influence multiple physiological functions and biological pathways in the pathophysiology of MASLD. In 2008, Horiguchi et al. (2008) identified HSD17B13 as a novel protein associated with lipid droplets, with expression predominantly in liver tissue, particularly within hepatic lipid droplets, which highlighted the potential significance of HSD17B13 in hepatic lipid metabolism and related diseases. Recent research suggested that SNPs in the HSD17B13 were critical for regulating hepatic lipid homeostasis, and may further have effect on the susceptibility and MASLD histological severity (Lin et al., 2020). Abul-Husn et al. (2018) performed an exome-wide association study using exome sequencing data and electronic health records from 46,544 European participants. This study identified the variant rs72613567 in HSD17B13 as a protective variant associated with lower serum transaminase levels (ALT and AST), reduced risks of MASH, and non-alcoholic cirrhosis. Notably, this variant was also linked to a decreased risk associated with the PNPLA3 I148M polymorphism. Interestingly, a study involving 111,612 participants from the Danish population found that individuals with a higher risk of fatty liver disease exhibited enhanced functionality of the HSD17B13 (rs72613567) variant in lowering ALT levels (Gellert-Kristensen et al., 2020a). Furthermore, another study by Kallwitz et al. (2020) found that the HSD17B13 rs72613567 variant was associated with a reduced risk of MASLD and a lower FIB-4 score in a cohort of 9,342 Hispanic or Latino individuals. A separate study involving a multi-ethnic Asian population of 165 individuals similarly observed that the HSD17B13 variants rs72613567 and rs6834314 were negatively correlated with the severity of MASLD and MASH, as well as with the degree of hepatocellular ballooning. These variants were also associated with a lower incidence of liver-related complications (Ting et al., 2021). Importantly, a recently study revealed HSD17B13 rs72613567-A variant protected against liver fibrosis by suppression of pyrimidine catabolism in humans and two different mouse models of MASLD (Luukkonen et al., 2023c).

Furthermore, numerous previous studies have indicated that SNPs within the GCKR gene are associated with MASLD (Zain et al., 2015). The GCKR gene plays a significant role in the negative regulation of glucose kinase activity (Agius, 2016). The SNP rs780094 in the GCKR is associated with hepatic lipid levels, and another SNP, rs1260326, diminishes the inhibitory effect on glucose kinase, prompting hepatocytes to increase glycolysis, which subsequently leads to hepatic steatosis. Yuan et al. (2022) conducted an association analysis involving 733 older Chinese individuals diagnosed with MASLD and 824 age- and race-matched controls. The variants rs780094 and rs1260326 of GCKR were found to be significantly associated with MASLD, with the T allele of rs1260326 correlating with elevated triglyceride levels. A GWAS involving 902 individuals with MASLD and 7,672 controls from the general population in Japan identified a significant association between the rs1260326 SNP in the GCKR gene and MASLD (Kawaguchi et al., 2018). In addition, they used the genetic variants of PNPLA3 (rs2896019), GCKR (rs1260326), and GATAD2A (rs4808199, rs17007417) to develop the risk-estimation models for MASLD and MASH, which is able to predict MASLD with higher accuracy and assist patients in making better treatment choices.

The SAMM50 gene encodes the Sam50 protein, which is located in the outer mitochondrial membrane and plays a critical role in the removal of reactive oxygen species, as well as maintaining mitochondrial morphology and division. Several studies have reported that the rs738491 variant in SAMM50 is an important SNPs that has a close association with MASLD in Japanese, Korean and Chinese Han papulation (Kitamoto et al., 2013; Chung et al., 2018; Xu et al., 2021). Li Z. et al. (2021) investigated the association between SAMM50 polymorphisms (rs738491 and rs2073082) and MASLD in a Chinese Han cohort, aiming to elucidate the functional implications of this relationship. The clinical data and corresponding blood samples were gathered from 380 individuals diagnosed with MASLD, along with 380 comparable control subjects. Through the case-control study, they found that individuals carrying the T allele of rs738491 or the G allele of rs2073082 had an increased risk of developing MASLD, which was associated with elevated triglyceride levels, ALT, and AST. Furthermore, they performed experiments in SAMM50 knockdown cells, and observed that SAMM50 gene variants reduced its expression. They also demonstrated that the impairment of SAMM50 function disrupted fatty acid oxidation (FAO), which led to lipid metabolic dysregulation in cells.

The MBOAT7 gene encodes the MBOAT7 protein, which functions as a lysophosphatidic acid acyltransferase (LPIAT), playing a crucial role in the reacylation of phospholipids within the phospholipid remodeling pathway (Johansen et al., 2016). In 2016, Mancina et al. (2016) carried out a GWAS in 1,149 individuals of European ancestry and pointed to a strong association between the rs641738 locus near the genes encoding MBOAT7-TMC4 and an increased risk of severe liver damage and fibrosis in MASLD patients. In accordance with human genetic research, cellular culture, and mouse models indicate that loss of MBOAT7 results in the accumulation of lipids in hepatocytes, exacerbating liver damage and promoting liver fibrosis (Mann et al., 2020; Thangapandi et al., 2021; Xia et al., 2021). In 2021 (Thangapandi et al., 2021), performed a knockout of the MBOAT7 gene in the liver of murine models. In hepatocytes with MBOAT7 deficiency, spontaneous steatosis occurred, which was characterized by elevated levels of cholesterol esters. After 6 weeks of feeding on a methionine-choline-deficient (MCD) diet, non-inflammatory fibrosis was observed in the MBOAT7 knockout mice. Furthermore, they genotyped the MBOAT7 variant (rs641738) in human liver biopsies and similarly found that this variant was associated with liver fibrosis (LF), independent of inflammatory processes. Importantly, Teo et al. (2021) conducted a meta-analysis using data from 1,066,175 participants across 42 European studies, and found that the rs641738 C>T mutation close to MBOAT7 gene was linked to liver fat, ALT and fibrosis in MASLD.

In addition to the aforementioned studies focusing on the comparatively well-characterized SNPs associated with lipid metabolism, several SNPs in other potential genes have also been implicated in lipid metabolic dysregulation in MASLD. Specifically, in humans, the G allele of rs174537 in the FADS1 gene was reported to be associated with increased desaturase activity of FADS1 as well as elevated levels of arachidonic acid in the bloodstream (Mathias et al., 2011). This association may be particularly pronounced in African American populations. The rs174616 variant in the FADS2 gene may affect fatty acid metabolism by inducing alterations in DNA methylation, which could potentially contribute to the pathogenesis of MASLD (Walle et al., 2019). GWAS have demonstrated a strong correlation between hepatic steatosis and the rs2228603 variant in the NCAN gene, as well as the rs4240624 variant in the PPP1R3B gene. Besides, the rs12137855 variation in the LYPLAL1 gene shows a significant association with histological features of MASLD (McCarthy et al., 2011). The FDFT1 gene is involved in the regulation of cholesterol synthesis, and the rs2645424 variant in FDFT1 has been associated with MASLD activity scores, as well as moderate to severe fibrosis in a cohort of overweight adolescents from diverse ethnic backgrounds (Santoro et al., 2013). A number of rare variants also predisposed individuals to MASLD and MASH. Specifically, rare mutations in the MTTP, APOB, and ATG7 genes have been associated with an increased susceptibility to MASLD. Interestingly, infrequent loss-of-function mutations in MTTP were known to induce abetalipoproteinemia (Chen et al., 2023b). The progression of the disease was exacerbated by the presence of rare loss-of-function mutations in the ATG7 gene, which disrupted lipophagy and mitophagy in hepatocytes (Moretti et al., 2024). Notably, a recent study found that rare protein-truncating variants in BSN were associated with an elevated risk of MASLD (Zhao et al., 2024).

It should be note that the prevalence of MASLD exhibits considerable variation across different racial and ethnic groups (Browning et al., 2004). Certain studies have aimed to validate the findings of the Genetics of Obesity-Related Liver Disease Consortium within diverse cohorts. Palmer et al. (2013) utilized computed tomography to assess hepatic steatosis in adult populations of African American and Hispanic descent. They found that the allele frequencies and effect sizes of the PNPLA3 rs738409, NCAN rs2228603, LYPLAL1 rs12137855, GCKR rs780094, and PPP1R3B rs4240624 variants differed among individuals of various racial backgrounds. In addition, hepatic steatosis showed a significant association with variants of PNPLA3, NCAN, GCKR, and PPP1R3B, along with nearby variants in African Americans. In Hispanic Americans, the associations were primarily observed with polymorphisms of PNPLA3 and PPP1R3B. Interestingly, a study conducted by Lin et al. (2014) evaluated the genetic diversity in obese children of Han Chinese descent in Taiwan, consisting of a cohort of 797 overweight or obese children aged 7–18 years. This study primarily focused on the impact of genetic variants PNPLA3 rs738409, NCAN rs2228603, LYPLAL1 rs12137855, GCKR rs780094, and PPP1R3B rs4240624 on MASLD. Their findings corroborated the association between the genetic variations GCKR rs780094 and PNPLA3 rs738409 with MASLD. Furthermore, they also discovered that NCAN rs2228603, LYPLAL1 rs12137855, and PPP1R3B rs4240624 were significantly linked to insulin resistance. On the other hand, the research indicated that genetic variations in GCKR and PNPLA3 may significantly increase the susceptibility of multiethnic obese individuals to MASLD. Collectively, genetic variants associated with lipid metabolism genes may elucidate the disparities in the incidence and mortality rates associated with MASLD, as well as its related conditions among individuals, families, and populations. All these findings enhance our understanding of the genetic basis of MASLD and MASH, while also providing a theoretical framework for the development of personalized treatment strategies.

## 4 The effect of DNA methylation in hepatic lipid-associated genes on MASLD and MASH

**FIGURE 3:**
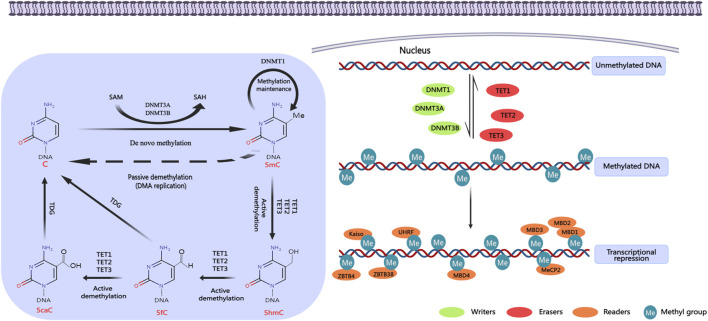
DNA methylation regulation. DNA methylation occurs mainly in the islands of cytosine-phosphate-guanine (CpG) gene promoter region. It promotes gene transcription in the promoter region by activating DNA methyltransferases. DNA methylases can be divided into three categories according to their roles in DNA methylation: writing enzymes, erasing enzymes, and reading enzymes. Writing enzymes catalyze the addition of methyl groups to cytosine residues. The function of erasing enzymes is to modify and remove methyl groups. Reading enzymes can recognize and bind methyl groups to affect gene expression. Created with MedPeer (medpeer.cn). C, cytosine; 5mC, 5-methylcytosine; 5hmC, 5-hydroxymethylcytosine; 5fC, 5-formylcytosine; 5caC, 5-carboxylcytosine; DNMTs, DNA methyltransferases; TETs, ten-eleven translocation enzymes; MBDs, methyl-CpG binding domain proteins; MeCP2, methyl CpG binding protein 2; UHRF, ubiquitin-like with PHD and RING finger domains; ZBTB4, zinc finger and BTB domain containing 4; ZBTB38, zinc finger and BTB domain containing 38.

DNA methylation represents a fundamental and ubiquitous modification found in eukaryotic cells, serving as the primary epigenetic mechanism regulating gene expression in mammals (Wu et al., 2023). This process facilitates the transmission of genetic information to progeny DNA through the action of DNA methyltransferases (DNMTs). Various forms of methylation modifications exist, including 5-methylcytosine (5mC), 5-hydroxymethylcytosine (5hmC), 5-formylcytosine (5fC), and 5-carboxylcytosine (5caC). Among these, 5mC is the most prevalent epigenetic modification within the human genome and has been extensively studied, conversely, the other forms of DNA methylation are comparatively rare. DNA methylases are classified into three distinct categories based on their specific functions in the DNA methylation process: writing enzymes, erasing enzymes, and reading enzymes (Figure 3) (Shi et al., 2022).

Writing enzymes facilitate the transfer of a methyl group from S-adenosylmethionine (SAM) to cytosine residues, primarily comprising members of the DNA methyltransferase (DNMT) family (Chen and Zhang, 2020). Within this family, DNMT3 is predominantly responsible for establishing methylation marks, whereas DNMT1 plays a crucial role in the maintenance of these methylation patterns. DNMT3 family encompasses enzymes DNMT3A, DNMT3B, DNMT3C, and DNMT3L, which facilitate the attachment of methyl groups to unmethylated cytosines. This process is known as *de novo* methylation (Zeng and Chen, 2019). DNMT3A and DNMT3B are the principal enzymes responsible for establishing DNA methylation during embryonic development (Bourc’his and Bestor, 2004). While DNMT3A and DNMT3B exhibit considerable structural similarity, they display different target preferences. DNMT3A is predominantly expressed during late embryonic development and in differentiated cells, whereas DNMT3B is mainly active during the early stages of embryonic development. Despite the fact that DNMT3L lacks intrinsic enzymatic activity, it serves as a critical cofactor in the *de novo* methylation process. Following the establishment of DNA methylation, DNMT1 exhibits a distinctive capability to replicate CpG methylation patterns and transfer them to the newly synthesized DNA strand. This process is essential for sustaining the DNA methylation status during DNA replication (Hyun and Jung, 2020).

The function of the erasing enzyme involves the modification and removal of methyl groups, with DNA demethylation occurring through either active or passive mechanisms. Active DNA demethylation is mediated by the TET (Ten−Eleven Translocation) enzymes, which includes TET1, TET2, and TET3 (Greenberg and Bourc’his, 2019). These enzymes catalyze the conversion of 5mC into 5hmC, 5fC, and 5caC. Subsequently, DNA demethylation is completed when thymine-DNA glycosylase (TDG) excises the base from 5fC and 5caC, thereby initiating the base excision repair pathway. In previous study, CpG methylation was considered to be a stable feature within the genomic context (Bird, 2002). However, the emergence of next-generation sequencing technologies allows for the measurement of DNA methylation at single-base resolution, which has uncovered that DNA methylation is highly dynamic, exhibiting alterations in response to variations within cellular and tissue microenvironments (Booth et al., 2012; Moore et al., 2013).

Reading enzymes are capable of recognizing and binding to methyl groups, thereby influencing gene expression, primarily manifested as transcriptional repression. This interaction encompasses three protein families that specifically recognize DNA methylation: Methyl-CpG binding domain (MBD) proteins, UHRF proteins, and zinc finger proteins (Shi et al., 2022). MBD proteins possess a MBD that demonstrates a high affinity for individual methylated CpG sites, with notable family members including MeCP2, MBD1, MBD2, MBD3, and MBD4. The UHRF protein family plays a critical role in the maintenance of DNA methylation by binding to DNMT1 and targeting hemimethylated DNA. In contrast, zinc finger proteins, such as Kaiso, ZBTB4, and ZBTB38, primarily exert transcriptional inhibition through mechanisms rely on DNA methylation.

In recent years, numerous studies have shown that the expression of lipid-related genes is closely associated with DNA methylation, as evidenced by both animal models and human liver biopsy samples. Abnormal methylation patterns of these genes are implicated in the mechanisms underlying the progression of MASLD and MASH, and they may also serve as potential biomarkers for evaluating the advancement of the disease.

### 4.1 Animal models

The dietary intake exerts a considerable impact on the pathogenesis of MASLD, therefor, the establishment of an optimal animal model for MASLD is essential for elucidating the etiology of this condition (Wolf et al., 2014; Liu et al., 2017; Li et al., 2021a; Li et al., 2021b). Nutrient imbalance models represent the primary approaches for the establishment of animal models relevant to MASLD and MASH. Researchers may develop animal model using various dietary interventions, including the western diet (WD), high-cholesterol diet (HCD), high-fat and high-fructose diet (HFHF), high-fat and high-cholesterol diet (HFHC), methionine and choline deficiency diet (MCD), and the choline-deficient, L-amino acid-defined high-fat diet (CDAHFD) (Oligschlaeger and Shiri-Sverdlov, 2020). Certain dietary components, such as betaine, folate, and choline, serve as methyl donors essential for the synthesis of S-adenosyl methionine (SAM). These methyl donors play a pivotal role in the metabolic pathways of methionine, in which SAM is integral to the process of genomic methylation (Niculescu and Zeisel, 2002; Kalhan et al., 2011). As a result, nutritional elements have emerged as critical determinants of DNA methylation (Table 2).

**TABLE 2:** List of the genes and the alteration of methylation involved in the development and progression of MASLD in mice livers.

Dietary model	Animal type	Phenotype	Methylation alterations	Reference
Methionine and choline deficiency model (MCD)	Wistar rats	Decreases: SAM/SAH ratio Increases: plasma homocysteine, free fatty acids, long-chain acylcarnitine		Sámano-Hernández et al. (2021)
Lack of methyl donor diet (lacking choline and folate)	C57BL/6J mouse	Reduced SAM	CpG island methylation of 164 genes (more hypomethylated than hypermethylated genes)	Tryndyak et al. (2010)
Betaine diet	C57BL/6J mouse	Improved HFD-induced hepatic steatosis	Hypo: Mttp promoter	Wang et al. (2014c)
Methyl donor diet (choline, betaine, vit. B12, folate)	Wistar rats	Improved HFS-induced hepatic steatosis	Hyper: Srebf2 (CpG23_24), Agpat3 (CpG10), Esr1 (CpG14)	Cordero et al. (2013)
Methyl donor diet (choline, betaine, vit. B12, folate)	Wistar rats	Improved HFS-induced hepatic steatosis	Hyper: Fasn (CpG10_11)	Cordero et al. (2012)
High-fat high-carbohydrate diet (HFHC)	C57BL/6J mouse	Decreases: methionine Increases: homocysteine	Hyper: Hmgcr	Pacana et al. (2015)
High-fat high-carbohydrate diet (HFHC)	C57BL/6J mouse	Downregulation: Sqle, Insig1, Hmgcr, Acsl1	Hyper: Sqle, Insig1, Hmgcr, Acsl1	Bi et al. (2023)
High-fat diet, high-fructose diet (HFD, HFrD)	C57BL/6J mouse	Weight gain and steatosis	Hypo: Apoa4, Atp1a1, Kcnj16, Nfatc1, Plb1 Hyper: Fgfr1, Ptpn11, Shank2, Gria1, Col4a2	Kim et al. (2019)
High-fat and high-sucrose (HF/HS)	Collaborative Cross (CC) mice	Severe susceptibility to MASLD	Hypo: Apoa4 Hyper: Gls2, Apom	Tryndyak et al. (2022)
High-fat diet (HFD)	C57BL/6J mouse	High DPP4 expression, hepatic steatosis, severe susceptibility to MASH	Hypo: Dpp4 (CpG877, CpG1204, CpG1253, CpG1255)	Baumeier et al. (2017)
Western diet (WD)	C57BL/6J (Apolipoprotein E-deficient mice)	Offspring with low ApoB expression	Hyper: ApoB gene promoter	Chen et al. (2020)
Resveratrol diet	C57BL/6J mouse	Low triglyceride levels and low expression of genes associated with lipogenesis	Hypo: Nrf2 gene promoter	Hosseini et al. (2020)

In 2021, Sámano-Hernández et al. (2021) established a MASLD model in rats through the administration of a methionine-choline deficient diet. For this MASLD mouse model, the ratio of S-adenosyl methionine to S-adenosyl homocysteine (SAM/SAH) exhibited a notable decline, while levels of plasma homocysteine, free fatty acids, and long-chain acylcarnitines were significantly elevated. The liver of mice subjected to a diet deficient in methyl donors such as choline and folate demonstrated diminished levels of S-adenosylmethionine (SAM). This finding was reported in a study led by (Tryndyak et al., 2010). Employing the CpG island microarray technique, they identified alterations in CpG island methylation across 164 genes within the liver tissue. These genes are associated with changes in lipid and glucose metabolism, DNA damage and repair, apoptosis, fibrosis progression, and liver tissue remodeling. Remarkably, the number of hypomethylated genes substantially outstripped that of genes undergoing hypermethylation. Betaine, a methylation donor, has been demonstrated to alleviate fatty liver induced by a HFD. This effect was achieved by reversing the hypermethylation of the Mttp promoter, which in turn ameliorated the Mttp dysregulation caused by the HFD (Wang et al., 2014c). This modification led to widespread hypomethylation across the genome, thereby promoting the export of triglycerides (TG) from the livers of mice subjected to a HFD. Consequently, there is a reduction in hepatic fat content, effectively mitigating hepatic steatosis. In another study, Cordero et al. (2013) utilized radiolabeled DNA synthesis to quantify total DNA methylation in a mouse model of MASLD induced by an HFS diet. To examine gene-specific methylation, a mass spectrometry technique known as EpiTYPER was employed in this study. Methyl-donor-supplemented with a dietary cocktail including folate, betaine, choline, and vitamin B12 was found to alter DNA methylation in the promoter regions of genes such as Agpat3, Esr1, and Srebf2, which are implicated in the pathogenesis of obesity and lipid metabolism. This regulatory mechanism plays a crucial role in restoring fat accumulation in the liver of rats subjected to HFS diets. This method also revealed that supplementation with methyl donors can induce hypermethylation of the Fasn gene, which probably had a significant role in the alleviation of MASLD due to the effects of methyl donor supplementation (Cordero et al., 2012).

In prevalent animal models associated with MASLD and MASH related to nutritional imbalances, Pacana et al. (2015) identified elevated homocysteine levels alongside methionine deficiency in the liver of mice with MASLD induced by a HFHC diet. The assessment of 5mC levels was conducted through the enzymatic breakdown of DNA into nucleotides. Although the overall DNA methylation remained unchanged, a significant downregulation of DNMT3A gene expression was observed in these mice. They employed methylation-specific restriction endonucleases to perform real-time quantitative PCR on the promoter CpG islands of critical genes, including Fasn and HMG-CoA reductase (Hmgcr). Notably, the Hmgcr gene exhibited substantial methylation levels. Recently, Bi et al. (2023) applied RNA sequencing (RNA-seq) and whole-genome bisulfite sequencing (WGBS) to investigate the role of DNA methylation and gene expression levels in a mouse model of MASH induced by a HFHC diet. Following integrated bioinformatic analysis, they identified that genes involved in cholesterol metabolism, including Sqle, Insig1, Hmgcr, and Acsl1, exhibited higher methylation levels coupled with lower mRNA expression. These findings indicated that alterations in DNA methylation within lipid metabolism genes as well as their related signaling pathways significantly contribute to the molecular events underlying MASH. Moreover, Kim et al. (2019) developed an animal model of MASLD by applying two distinct dietary interventions to C57BL/6J mice: HFD and a high-fructose diet (HFrD). They conducted a genome-wide methylation analysis utilizing reduced representation bisulfite sequencing (RRBS-seq) for liver DNA. Based on KEGG pathways analyses for the differentially methylated CpGs, they identified genes such as Apoa4, Atp1a1, Kcnj16, Nfatc1, and Plb1 predominantly displayed sustained hypomethylation. Conversely, genes including Fgfr1, Ptpn11, Shank2, Gria1, and Col4a2 were primarily associated with sustained hypermethylation. Notably, the researchers emphasized the relationship between Apoa4 and liver triglyceride exportation, highlighting its potential role in metabolic regulation in MASLD. Additionally, Tryndyak et al. (2022) observed distinct differences in DNA methylation and gene expression levels of the Apoa4, Gls2, and Apom genes in mice predisposed to severe MASLD. These differential patterns were not evident in mice exhibiting mild MASLD phenotypes. The analysis was conducted using genome-wide targeted bisulfite DNA methylation next-generation sequencing on liver tissues obtained from Collaborative Cross (CC) mice subjected to a high-fat and high-sucrose (HF/HS) diet. Although the two studies employed different dietary interventions to establish animal models of MASLD, both independently identified alterations in the methylation status of the Apoa4 gene within these models. Apolipoprotein A-IV (Apoa4) plays a critical role by binding to chylomicrons to facilitate the transport of dietary lipids. Additionally, Apoa4 is involved in the formation and remodeling of high-density lipoprotein (HDL), which aids in the reverse transport of cholesterol and enhances the clearance of excess cholesterol from peripheral tissues. Consequently, changes in the expression levels and DNA methylation of the Apoa4 gene provide valuable insights into the mechanisms underlying the pathogenesis of MASLD. These findings suggest that alterations in the expression and DNA methylation status of lipid-related genes may serve as predictive markers for the development of MASLD.

Dipeptidyl peptidase 4 (DPP4) is an adipokine secreted by hepatocytes, and expression of hepatic Dpp4 are elevated in individuals who are overweight or have MASLD. In a study involving mice fed a HFD for 6 weeks, liver DPP4 expression was found to increase concomitantly with weight gain. Baumeier et al. (2017) conducted their investigation using direct bisulfite sequencing PCR (dBSP) and pyrosequencing to examine methylation profile of Dpp4 gene in mouse liver. They identified reduced methylation at four CpG sites within the DPP4 gene in HFD mice. Furthermore, analysis of liver biopsy samples from overweight individuals also revealed that elevated hepatic Dpp4 levels were associated with lower DNA methylation as specific CpG site in both hepatosteatosis and MASH. In a study conducted by Chen et al. (2020), apolipoprotein (Apo) E-deficient female mice were fed a WD characterized by elevated levels of fat and cholesterol. The researchers investigated the implications of this dietary exposure on the development of MASLD in male offspring of WD-fed dams. Their findings revealed that male offspring exhibited lower serum levels of Apolipoprotein B (ApoB) alongside reduced hepatic expression of the ApoB gene. Subsequent DNA methylation analysis was performed using bisulfite sequencing, indicated that the livers of male offspring from dams fed a WD displayed increased methylation within the promoter region of the ApoB gene. These results suggested that maternal consumption of a western diet may induce epigenetic modifications that alter ApoB gene expression, thereby exacerbating the risk of MASLD in male offspring. Nuclear factor erythroid 2-related factor 2 (NRF2) is a transcription factor that plays a protective role against MASLD by downregulating the expression of genes implicated in lipid accumulation. Reduced expression and activity of NRF2 have been documented in livers of MASH. In a study involving mice subjected to a HFD, resveratrol was shown to induce hypomethylation of the hepatic Nrf2 promoter. This hypomethylation was associated with decreased triglyceride levels and reduced expression of lipogenic genes, such as fatty acid synthase (Fasn) and sterol regulatory element-binding protein 1c (Srebp-1c) (Hosseini et al., 2020).

Taken together, in various animal models of MASLD and MASH induced by dietary modifications, alterations in the methylation patterns of hepatic genes associated with lipid metabolism have been observed. These findings suggest that environmental factors, such as diet, may influence the expression of genes related to lipid metabolism through epigenetic mechanisms, particularly via abnormal changes in DNA methylation.

### 4.2 Human studies

Abnormal DNA methylation patterns have been associated with dysregulated gene expression and are implicated in the pathogenesis of various human diseases (Zeybel et al., 2015; Pardo et al., 2022; Kang et al., 2023; Wu et al., 2023). With the continuous advancement of DNA methylation detection methods and the in-depth investigation of DNA methylation changes in the process of MASLD, researchers have obtained substantial information regarding specific differential DNA methylation in patients with varying stages of MASLD (Supplementary Table S5) (Sookoian et al., 2009; Murphy et al., 2013; Pirola et al., 2013; Nishida et al., 2016; Baumeier et al., 2017; Nano et al., 2017; Tian et al., 2020; Yaskolka Meir et al., 2021; Pan et al., 2022; Magdy et al., 2024). These global hypomethylation and specific differential methylation events play a crucial role in the progression of MASLD, particularly the methylation modifications occurring at the transcriptional start sites of genes involved in lipid metabolism (Table 3) (Kitamoto et al., 2015; Lai et al., 2019; Ma et al., 2019; Walle et al., 2019; Motta et al., 2023). These methylation alterations in lipid metabolism-related genes will provide valuable insights into the mechanisms underlying the occurrence and progression of MASLD.

**TABLE 3:** List of the lipid metabolism-related genes and the alteration of methylation involved in the development and progression of MASLD in human liver and blood.

Gene name (CpG site)	Sample type	Correlations with DNA methylation	Number of subjects	DNA methylation measurement Method(s)	Reference
Hyper: PPARGC1A (promoter)	Liver biopsy	Plasma fasting insulin level, HOMA-IR	11 Controls, 63 MASLD	MS-PCR	Sookoian et al. (2010b)
Hyper: PNPLA3 (CpG_99)	Frozen liver biopsy	Liver fibrosis, rs738409-CpG_99	65 MASLD	Targeted-bisulfite sequence analysis	Kitamoto et al. (2015)
Hypo: PDGFRA (promoter CpG_3) Hyper: PPARA (promoter CpG_3), PPARD (promoter CpG_2)	Paraffin-embedded liver biopsy	Liver fibrosis	17 MASLD, 10 ALD	Bisulfite pyrosequencing	Zeybel et al. (2015)
Hypo: PRKCE (cg04035064), IP6K3 (cg10714061), ACLY (cg25687994), PLCG1 (cg18347630)	Frozen liver biopsy	MASLD	18 Controls, 45 Obesity	Infinium Human Methylation 450K BeadChip Bisulfite sequencing	Ahrens et al. (2013)
Hyper: EPHX1 (cg26187962, cg03337430, cg24928687, cg17468616, cg23096144, cg03459809, cg05385434, cg25152404, cg24868305), SLC51A (cg17526770, cg27558485, cg08877188, cg21748136, cg05473677, cg04478991), SLC10A1 (cg05633152, cg21088438, cg01448863), SLC27A5 (cg19469742, cg18495710, cg16107172, cg07726085, cg16278661, cg06621784), SLCO2B1(cg12537437, cg25367084, cg23577865, cg20358275, cg15751948, cg18589858)	Liver biopsy	MASLD, liver fibrosis	75 Controls, 103 MASLD	Infinium Human Methylation 450K BeadChip	Schiöth et al. (2016)
Hyper: SCD (cg02237755, cg24503796, cg03440556, cg06400428, cg18328965), NPC1L1 (cg06907626, cg14477619, cg12252759, cg05358848, cg23118549), G6PC1 (cg05353659), FGF21 (cg13881341, cg21107581, cg21967668), APOC4 (cg17769836, cg25017250,cg04401876, cg06736138, cg27353824), APOB (cg00673290, cg26112457, cg07636176, cg25035485), APOA2 (cg18281418, cg01053621, cg08922317), ACOX2 (cg16587010, cg22012981, cg16209444)	Liver biopsy	MASLD, liver fibrosis	75 Controls, 103 MASLD	Infinium Human Methylation 450K BeadChip	Mwinyi et al. (2017)
Hypo: HDAC9 (cg16925459), SLCO3A1 (cg03756778), BMP2 (cg16831623) Hyper: C1QTNF1 (cg00193613)	Liver biopsy	MASH, serum fasting insulin level	95 Obesity	Infinium Human Methylation 450K BeadChip	de Mello et al. (2017)
Hypo: FADS2 (cg06781209, cg07999042)	Liver biopsy	D6D activity, rs174616 -cg07999042	95 Obesity	Infinium Human Methylation 450K BeadChip	Walle et al. (2019)
Hypo: CCN1, PDGFA, AQP1, PIP4P2 Hyper: ETNK2, NEU4, RBP5	Frozen liver biopsy	Liver fibrosis	35 Mild MASLD 25 Advanced MASLD	Infinium Human Methylation 450K BeadChip	Hotta et al. (2018)
Hypo: NSMAF, ANXA2 Hyper: BIN1, EPHX2, ACADS, LSS, APOM	Liver biopsy	Liver fibrosis	15 Controls, 14 MASLD	Infinium Human Methylation 450K BeadChip	Gerhard et al. (2018)
Hypo: ACSL4 (cg15536552, cg06822229), CPT1C (cg21604803)	Peripheral blood	Risk for MASLD	30 Controls, 35 MASLD	Infinium Human Methylation 450K BeadChip Bisulfite pyrosequencing	Zhang et al. (2018)
Hyper: ABCC1 (cg04981696)	Peripheral blood	MASH specific	30 Controls, 35 MASLD	Infinium Human Methylation 450K BeadChip Bisulfite pyrosequencing	Wu et al. (2018)
Hypo: DHCR24 (cg17901584), MSMO1 (cg05119988), CPT1A (cg00574958) Hyper: SREBF1(cg11024682), ABCG1 (cg27243685, cg06500161)	Peripheral blood	Hepatic fat	3400 European ancestry, 401 African ancestry, 724 Hispanic ancestry	Infinium Human Methylation 450K BeadChip	Ma et al. (2019)
Hyper: PPARG (CpG_1, CpG_2)	Plasma	Liver fibrosis	9 Controls, 26 MASLD, 13 ALD cirrhosis	Bisulfite pyrosequencing	Hardy et al. (2017)

#### 4.2.1 DNA methylation alterations in liver tissue

Currently, liver biopsy remains the gold standard for diagnosing MASLD and MASH, providing the most accurate assessment of inflammatory damage and fibrosis staging in hepatic tissue (Leng et al., 2021). To investigate the changes in site-specific DNA methylation of lipid metabolism-related genes in the liver tissues of MASLD, researchers utilized Methylation Specific Polymerase Chain Reaction (MS-PCR) to obtain methylation information at specific position (Sookoian et al., 2010b). A study that involved DNA methylation analysis using MS-PCR on liver biopsy samples from 18 control participants and 47 patients with MASLD. The results revealed a higher methylation level of PPARGC1A promoter in MASLD livers compared to control livers, along with a lower mRNA level of PPARGC1A. In another study, Kitamoto et al. (2015) performed a target-bisulfite sequencing to explore the methylation status of four different CpG islands (CpG99, CpG71, CpG26, and CpG101) within the regulatory regions of the PNPLA3, SAMM50, and PARVB. This study found that CpG26 in the regulatory region of PARVB variant 1 was significantly hypomethylation, and CpG99 of PNPLA3 were remarkably hypermethylation in the livers of patients with advanced MASLD relative to those with mild MASLD, respectively. Notably, a significant negative correlation was observed between the abundance of PNPLA3 mRNA and CpG99 methylation levels in MASLD patients. These findings suggested that the abnormal DNA methylation of PNPLA3 and PARVB may play a crucial role in the fibrosis severity in patients with MASLD or chronic hepatitis C infection. In addition, bisulfite pyrosequencing has emerged as a powerful technique for analyzing DNA methylation due to its high sensitivity and specificity. Zeybel et al. (2015) examined the methylation status of paraffin-embedded liver biopsy specimens from 17 patients with MASLD using bisulfite pyrosequencing. Among these patients, eight had mild fibrosis, while nine exhibited severe fibrosis. The results demonstrated that those with severe MASLD showed increased methylation of specific CpG sites in the promoters of PPARα (CpG3) and PPARγ (CpG2). In contrast, these patients had low methylation levels of specific CpGs in the promoters of PDGFα (CpG3).

Since Illumina developed a platform for whole-genome DNA methylation analysis at single CpG resolution, which can simultaneously detect millions of methylation sites, and this method has also been widely used to investigate the specific DNA methylation changes associated with MASLD (Ammerpohl et al., 2011; Ahrens et al., 2013; Kuramoto et al., 2017; Mwinyi et al., 2017). Ahrens et al. (2013) conducted a study using the Infinium HumanMethylation 450 BeadChip and bisulfite sequencing to analyze DNA methylation patterns and mRNA expression profiles in various liver samples. Those individuals included 18 normal controls, 18 healthy obese, 12 steatosis, and 15 MASH. The results revealed that four genes exhibiting low methylation levels (ACLY, PLCG1, PRKCE, and IP6K3) are closely associated with the pathogenesis and progression of MASLD. Notably, these genes encode key enzymes that play critical roles in lipid metabolism and signal transduction. For instance, ACLY (ATP-citrate lyase) catalyzes the conversion of citrate to acetyl-CoA, thereby linking glucose metabolism to fatty acid synthesis, while PLCG1 (phospholipase C gamma 1) promotes the production of diacylglycerol and inositol trisphosphate (IP3) (Pirola and Sookoian, 2020). In another study, multiple CpG site DNA methylation analysis was carried out using the Infinium HumanMethylation 450 BeadChip on 103 patients with MASLD and 75 non-MASLD patients to investigate the methylation and transcriptional changes of bile acid homeostasis and drug metabolism genes in MASLD. They found that, in comparison with non-MASLD patients, MASLD patients exhibited several differential methylation sites in lipid metabolism-related genes, such as EPHX1, SLC27A5, SLC51A, SLC10A1, and SLCO2B1, which displayed a hypermethylated state and lower mRNA expression levels (Schiöth et al., 2016). A year later, Mwinyi et al. (2017) from the same team utilized the same database to gain further insights into the methylation changes of lipid metabolism genes. They investigated a cluster of 74 lipid metabolism-related genes and identified 41 genes with significant methylation differences associated with the progression of MASLD. Notably, specific CpG loci in the SCD, ACOX2, APOC4, APOB, FGF21, APOA2, NPC1L1, and G6PC exhibited a high methylation status that was closely related to their transcriptional expression changes during the progression of MASLD. In another study involving 26 patients with MASH, 35 patients with simple fatty liver, and 34 individuals with normal liver phenotypes, significant DNA methylation-specific changes were identified at 1,292 CpG sites across 677 genes in the MASH liver biopsy samples. Among these, 30 genes exhibited DNA methylation sites that was associated with their mRNA expression. They detected the hypomethylated CpGs of lipid metabolism-related genes, including HDAC9, SLCO3A1, and BMP2, while the CpGs mapped to C1QTNF1 was hypermethylated (de Mello et al., 2017). Interestingly, based on the published methylation dataset of these 95 obese individuals, Walle et al. (2019) made new discoveries regarding FADS2, which encodes delta-6 desaturase (D6D), an enzyme essential for the metabolism of polyunsaturated fatty acids (PUFAs). Previous studies have already established a positive correlation between MASH and the expression levels of FADS2 in liver tissue (Walle et al., 2016). Subsequently, Walle et al. (2019) explored the relationship between methylation changes of FADS2 and its expression levels in the 95 obese individuals. The findings revealed a negative correlation between the methylation levels of two specific CpG sites associated with the FADS2 gene (cg07999042 and cg06781209) and with the activity of delta-6 desaturase (D6D) on the base of both liver and serum fatty acids. Among them, the methylation level of FADS2 (cg07999042) is closely associated with the FADS2 variant rs174616. Moreover, by analyzing the DNA methylation profiles of MASLD patients, low methylation and high expression levels were observed for the liver lipid metabolism-related genes CCN1, PDGFA, AQP1, and PIP4P2 in cases of severe MASLD. Conversely, high methylation and low expression levels were found in ETNK2, NEU4, and RBP5 (Hotta et al., 2018). In the same year, Gerhard et al. (2018) analyzed the whole-genome methylation profiles of age- and sex-matched control patients (n = 15) and patients with stage 3/4 MASLD (n = 14) who exhibited histological liver phenotypes, aiming to explore the methylation characteristics associated with MASLD-related cirrhosis. The study identified 99 hypomethylated and 109 hypermethylated CpG islands, including 34 sites negatively correlated with liver gene expression. Among the differentially methylated genes, the hypomethylated lipid metabolism-related genes were NSMAF and ANXA2, while hypermethylated lipid metabolism-related genes included BIN1, EPHX2, ACADS, LSS, and APOM.

Increasing evidence indicated that DNA methylation could act as a predictive biomarker for the risk of liver cancer development in patients with MASLD and MASH. The methylation levels of individual or multiple lipid-related genes may serve to evaluate the risk of MASLD and MASH. These findings not only deepen our understanding of the underlying mechanisms of these diseases but also highlight the important clinical implications of DNA methylation for diagnosis, prognosis, and potential therapeutic interventions for MASLD patients in the future. Such insights could lead to more personalized approaches to treatment and monitoring, ultimately improving patient outcomes.

#### 4.2.2 DNA methylation alterations in peripheral blood

Performing liver biopsies to reliably confirm MASH and to measure gene expression alongside DNA methylation is a highly invasive procedure, which carries risks for patients. As a more accessible alternative, the assessment of DNA methylation in peripheral blood mononuclear cells (PBMCs) is emerging as a potential biomarker for the diagnosis of MASLD (Hardy et al., 2017; Loomba et al., 2018; Ma et al., 2019). The research teams conducted an epigenome-wide association studies (EWAS) using this minimally invasive method to detect DNA methylation changes in PBMCs of patients with varying stages of MASLD. The aim is to determine whether specific methylation changes can facilitate the stratification of patients with MASLD who are at a higher risk of liver fibrosis. In fact, as early as 2013, a study developed an age predictor (Hannum Epigenetic Clock) based on the analysis of DNA methylation changes in peripheral blood (Hannum et al., 2013). This predictor utilizes information derived from 27 CpG sites to estimate biological age. Although the initial purpose of epigenetic clocks was to estimate chronological age, research has indicated that the discrepancy between chronological age and epigenetic age serves as a significant predictor for complex diseases (Margiotti et al., 2023). Loomba et al. (2018) employed the Horvath Epigenetic Clock to assess the methylation levels of 353 age-related CpG sites in the peripheral blood of MASH patients, which included 193 hypermethylated and 160 hypomethylated CpGs. The results indicated that MASH patients at fibrosis stages F2-F3 exhibited accelerated epigenetic aging compared to the control group.

Regarding the DNA methylation changes in peripheral blood leukocytes during the progression of MASLD, we are particularly interested in the alterations associated with lipid metabolism-related genes. Zhang et al. (2018) carried out an EWAS of peripheral blood leukocytes from 35 individuals with MASLD and 30 healthy controls from the Han Chinese population. Utilizing the 450K BeadChip and bisulfite pyrosequencing for methylation profiling, they identified methylation alterations at 863 different CpG sites in MASLD patients, primarily characterized by global hypomethylation. The findings revealed a heightened risk of MASLD associated with low methylation levels at specific CpG sites in the ACSL4 gene (cg15536552) and the CPT1C gene (cg21604803), both of which are significant in adipocyte signaling pathways. In the same year, Wu et al. (2018) conducted a comparison of global methylation levels among groups with simple steatosis, MASH, and healthy controls using the same sample sets and methodologies. In contrast to the healthy control group, 35 patients with MASLD exhibited methylation changes at 65 CpG sites associated with 60 genes in circulating blood leukocytes. Within the simple steatosis group, 32 CpG sites were correlated with levels of triglycerides (TG), total cholesterol (TC), or low-density lipoprotein cholesterol (LDL-C), indicating the presence of dyslipidemia. Among these 32 sites, 11 were significantly associated with the histological features of MASLD. Notably, the ABCC1 gene (cg04981696) was identified as the only lipid metabolism-related gene linked to hepatic steatosis. In another study involving peripheral blood samples from 3,400 participants of European ancestry, 401 participants of Spanish ancestry, and 724 participants of African ancestry, the researchers performed a comprehensive epigenome-wide association analysis to explore the relationship between DNA methylation at over 400,000 CpG sites and LFC. Among the participants of European descent, the methylation levels of 22 CpG sites were associated with liver fat. Specifically, the hypermethylated CpGs mapped to lipid metabolism-related genes included DHCR24 (cg17901584), SC4MOL (cg05119988), and CPT1A (cg00574958), while the hypomethylated CpGs loci in lipid metabolism-related gene comprised SREBF1 (cg11024682) and ABCG1 (cg27243685, cg06500161) (Ma et al., 2019).

In addition to the assessment of DNA methylation in PBMCs, dying hepatocytes release degraded genomic DNA into the circulation, resulting in the easy acquisition of this circulating free DNA (cfDNA) from plasma (Moran-Salvador and Mann, 2017). Given that hepatocyte death is a primary pathological feature of MASLD, quantifying the methylation of cfDNA in the plasma of patients may reflect the severity of MASLD and is proposed as a high-precision alternative for MASLD stratification (Hardy et al., 2017). A study conducted in 2016 utilized bisulfite pyrosequencing to investigate the methylation changes of circulating DNA in the plasma of MASLD patients (Hardy et al., 2017). The findings unveiled that the DNA methylation level of the PPARγ gene promoter increased with the severity of fibrosis associated with MASLD. Based on the methylation levels of the differentially methylated regions (DMRs) within the PPARγ gene promoter, plasma DNA can be analyzed to non-invasively stratify the risk of fibrosis in MASLD. This liquid biopsy biomarker has the potential to serve as an important clinical tool. We require more extensive and in-depth studies to establish a comprehensive database of liver and hepatocyte-specific DNA methylation patterns. This would enhance the accuracy and sensitivity of cfDNA as a potential biomarker for MASLD.

## 5 The relationship between genetic variants and DNA methylation of lipid metabolism–related genes and immune response in MASLD and MASH

An increasing number of studies have demonstrated that the immune pathways are crucial in the pathogenesis of MASLD and MASH (Parthasarathy et al., 2020; Wang et al., 2023a), highlighting the potential involvement of MASLD and MASH-related risk variants and DNA methylation mediating the immune-driven disease severity (Sookoian and Pirola, 2023; Zhang et al., 2023). Although hepatic immune cells are critical in the pathogenesis of different liver disease including MASH (Guilliams and Scott, 2022), presently, there is a absence of extensive research directly connecting the genetic predisposition of genes like PNPLA3, TM6SF2, MBOAT7 and HSD17B13 to immune regulation in the context of MASH. Nevertheless, certain conclusions can be extrapolated based on their established cellular functions. For instance, a recent study suggested that PNPLA3 I148M macrophages exhibit a proinflammatory phenotype, exacerbating lipid metabolism dysregulation in MASLD (Dixon et al., 2023). More specifically, compared to wild-type PNPLA3 hepatic stellate cells (HSCs), HSCs carrying PNPLA3 I148M showed upregulated secretion of cytokines, including CCL5, GM-CSF, and CXCL8. When THP-1-differentiated macrophages were treated with conditioned media from PNPLA3 I148M HSCs or wild type PNPLA3 HSCs, they displayed a pronounced chemotactic response (Bruschi et al., 2017). Notably, Kabbani et al. (2022) established a human pluripotent stem cell (hPSC)-derived multicellular liver culture by incorporating hPSC-derived hepatocytes, HSCs, and macrophages to model MASLD. They generated an isogenic pair of hPSCs harbouring wild-type (WT) or I148M PNPLA3, they did not discover cell-intrinsic differences in hPSC^*I148M*^-derived hepatocytes, macrophages, and HSCs. In their liver cultures, PNPLA3 was identified in HSCs and hepatocytes, but not in macrophages as previously reported (Pirazzi et al., 2014). Then, they compared the response of PNPLA3^*WT*^ or PNPLA3^*I148M*^ liver cultures after 2 weeks in lipotoxic conditions, they found that PNPLA3^*I148M*^ HSCs show enhanced and accelerated activation under prolonged lipotoxic insult. Most importantly, they observed an elevated IL-6/STAT3 activity induced by NF-κB activation in PNPLA3^*I148M*^ liver cultures. By comparing the purified cells from PNPLA3^*WT*^ and PNPLA3^*I148M*^ cultures, the found the elevated IL-6 expression comes primarily from macrophages. In another recently study, Kabbani et al. (2022) developed an animal model to investigate the role of human hepatocytes in MASLD, after feeding huFNRG mice on western diet (WD) for 4 weeks, they found inflammatory infiltrates were detected in a subset of huFNRG mouse, including an increasing number of hepatic macrophages as well as neutrophils. Interestingly, in contrast to previous 148I-huFNRG studies, PNPLA3 148M-huFRG livers contained high grade of ballooning degeneration and lobular inflammation. These data suggested that there are potential links between PNPLA3 variant and macrophage function in liver cultures and mouse model. Interestingly, recent studies showed that the PNPLA3 I148M variant alters HSCs biology via attenuation of PPARγ, AP-1, LXRα and TGFβ activity (Pingitore et al., 2016; Bruschi et al., 2017; Bruschi et al., 2019). Additionally, other studies have shown an association between PNPLA3 rs738409 and serum levels of soluble intercellular adhesion molecule 1 (sICAM-1), an inflammatory marker produced by endothelial and immune cells (Georges et al., 2011). Research indicates that hepatic ICAM-1 expression levels are significantly correlated with the severity of lobular inflammatory infiltration and necroinflammatory activity (Sookoian et al., 2010a). In addition (Sookoian and Pirola, 2023), retrieved from the PhenoScanner database a set of SNPs that induce changes in immune-related gene expression in blood. The PNPLA3 rs738409 variant also correlates with whole-blood expression of FAM89B, which negatively regulates TGFβ-induced signaling—a critical pathway in the immune response (Batlle and Massagué, 2019). Moreover, the rs58542926 variant in TM6SF2 has been linked to blood expression levels of CXCL9, a chemokine superfamily member that encodes a secreted protein involved in immune regulation and inflammation (Kitade et al., 2017). Lastly, rs641738 in MBOAT7 has been associated with whole-blood levels of LILRP1 (leukocyte immunoglobulin-like receptor pseudogene 1). These findings collectively underscore the intricate links between genetic factors, immunological pathways, and the progression of MASH and related liver diseases. In addition, polymorphisms rs72613567:TA in HSD17B13 have been shown to confer protection from liver inflammation in chronic liver disease (Abul-Husn et al., 2018; Luukkonen et al., 2020), by regulating lipid accumulation and pyrimidine catabolism (Su et al., 2014; Luukkonen et al., 2023c). However, the specific mechanism by which human HSD17B13 may regulate inflammation through metabolic enzyme activity was rarely reported. It has been reported that HSD17B13 liquid–liquid phase separation promotes leukocyte adhesion in chronic liver inflammation (Ye et al., 2024). It should be note that single-cell RNA-seq (scRNA-seq) analysis showed that human HSD17B13 is mainly localized in hepatocytes, with very low expression in other liver cells such as cholangiocytes, macrophages, hepatic stellate cells, liver sinusoidal endothelial cells (LSECs), T cells, and plasma cells (MacParland et al., 2018; Sookoian and Pirola, 2023). Similarly, the mouse HSD17B13 gene is also mainly expressed in mouse hepatocytes, with very low levels in Kupffer cells, LSECs, B cells and NK cells (Schaum et al., 2018). Although the effects of HSD17B13 epigenetic modifications on pro-inflammatory cytokine networks or hamper immune-driven fibrotic pathways are still unclear, the application of scRNA-seq in future research may shed light on the epigenetic regulation of HSD17B13 in the immune microenvironment of MASLD and MASH.

Moreover, various T-cell subtypes have been identified as significant contributors to the development of MASH and its potential transformation into HCC. A current summary of the intricate involvement of innate and conventional T cells in MASH is detailed by (Hirsova et al., 2021). Investigations across human and mouse experimental systems revealed that CD8 T cells facilitate MASH progression through the production of pro-inflammatory mediators and non-specific hepatocellular cytotoxicity (Dudek et al., 2021; Koda et al., 2021). In addition, recent work have underscored the importance of TREM2+ macrophages in MASH pathogenesis. A study conducted by Fredrickson et al., reported that TREM2+ macrophages may exert a beneficial effect in the context of MASH and bariatric surgery (Fredrickson et al., 2024). Another group reported that TREM2+ macrophages restrain MASH pathology, as well as facilitate MASH with fibrosis resolution, by attenuating inflammasome activation and tissue inflammation while promoting phagocytosis, ECM degradation, and lipid metabolism (Ganguly et al., 2024). These findings highlight the need to further explore how alterations in DNA methylation might intersect with immune mechanisms in MASH. It has been reported that pharmacological inhibition of PPARγ1 promoter DNA methylation via 5-aza-2′-deoxycytidine or genetically by DNMT1 knockout promotes macrophage alternative activation in obesity (Wang et al., 2016). A study by Zhou et al. (2021) in mice demonstrated that knockdown of the Osr1 gene led to changes in the expression of Ccl3 and Pcgf2, along with alterations in CpG methylation sites. These molecular modifications coincided with increased macrophage infiltration and inflammation in the liver. Honggui Li’s group recently examined the role of hepatic adenosine kinase (ADK) in the context of fat accumulation and liver inflammation (Li et al., 2023a). ADK is an enzyme that phosphorylates adenosine to adenosine monophosphate and promotes the methionine cycle, thereby enhancing methylation reactions. They found that hepatic ADK levels were abnormally elevated in MASLD patients. Moreover, liver-specific ADK overexpression in mice resulted in weight gain, increased obesity, and more severe hepatic steatosis and inflammation. Mechanistically, ADK upregulates DNA methylation and suppresses Ppara, thereby promoting lipid deposition in hepatocytes. Furthermore, ADK in hepatocytes drives the proinflammatory activation of liver nonparenchymal cells via ADK-driven hepatocyte mediators, involving macrophage STING and Ly6c2. Given the link between DNA methylation and immune pathways in MASLD progression, Pant et al. (2023) employed a DNA demethylating agent (a DNMT1 inhibitor) to treat MASLD mice fed a Western-style diet. They discovered that, in the treated MASLD mice, DNA methylation was reduced at the promoters of autophagy-related genes in hepatic macrophages, leading to elevated gene expression. This shift drove macrophage polarization toward the M2 phenotype, subsequently mitigating inflammation and halting MASLD progression. Moreover, a recently study on DNMT1 gene knockout (LD1KO) mice showed that DNMT1 deficiency ameliorated HFD-induced hepatic steatosis in mice. To elucidate the cell type-resolved mechanisms by which DNA methylation regulates hepatic lipid metabolism, they conducted single-nucleus RNA sequencing (snRNA-seq) on frozen liver specimens from liver-specific deletion of Dnmt1 mice and their fl/fl controls using 10X genomics sequencing methodology. They discovered a significant reduction in macrophages/Kupffer cells, declining from 25% in fl/fl liver to 9.4% in LD1KO mice, coinciding with a concurrent decrease in other immune cell populations, including T cells (from 5.3% to 3.2%), dendritic cells (from 2% to 1%), and endothelial cells (from 37.5% to 28.8%). Their results suggested that Dnmt1 deletion prevents HFD-induced remodeling of the liver cell compositions by reducing immune cell migration into hepatic tissue, consequently preserving liver homeostasis (Wang et al., 2023b). These findings collectively underscore the importance of epigenetic regulation in MASLD pathogenesis and pave the way for further exploration of DNA methylation–targeted therapies.

## 6 The interplay between extrahepatic signals from other tissues and DNA methylation of lipid metabolism–related genes in MASLD and MASH

MASLD is regarded as a systemic disease characterized by intricate crosstalk among adipose tissue, muscle, and the gut–liver axis (Fan et al., 2025). For instance, adipose tissue, serving as a key organ for interorgan communication, releases a variety of signaling molecules that affect energy balance and immune regulation (Ghesmati et al., 2023). The infiltration of macrophages producing inflammatory cytokines in adipose tissue significantly aggravates inflammatory processes in liver (Bijnen et al., 2018). Specially, resistin, predominantly produced by white adipose tissue (WAT) in mice and observed in human preadipocytes and mature adipose tissues, is associated with insulin resistance (IR) and contributes to MASLD (Filková et al., 2009). Notably, gut microbiota–induced DNA methylation changes may play a particularly important role in the pathogenesis of MASLD (Tang et al., 2023). Research has shown that antibiotic-mediated gut microbiome modulation prevents diet-induced weight gain and adipocyte expansion. The reduction of *Firmicutes*, *Lactobacillus*, and *Helicobacter*, along with the increase in *Bacteroides*, *Enterobacter*, and *Klebsiella*, enhances adipose tissue expression of adiponectin and resistin through promoter DNA hypomethylation and downregulation of DNMT1 and DNMT3A (Yao et al., 2020). Another study has shown that the gut microbiome can maintain intestinal immune homeostasis by altering Toll-like receptor 4 (TLR4) DNA methylation in intestinal epithelial cells (IECs), achieved through DNMT3 recruitment, a mechanism crucial to hepatic fibrosis and steatosis progression (Narabayashi et al., 2022). Moreover, short-chain fatty acids (SCFAs), which are the principal metabolites produced by microbial fermentation of indigestible carbohydrates, can directly reduce the expression of DNMT1, DNMT3a, and DNMT3b and inhibit their binding to the promoters of adiponectin and resistin, thereby correcting the abnormal expression of these molecules in obesity (Lu et al., 2018). On the other hand, the gut microbiome serves as an important source of vitamins, including vitamin B9 (folate) and vitamin B12 (cobalamin). Acting as key methyl donors, these vitamins modulate one-carbon metabolism, thereby influencing DNA methylation in MASLD (Abe et al., 2021). In the brain, specific nuclei and neural networks integrate crucial metabolic hormones and neuropeptides from peripheral sources, facilitating adaptive adjustments of food intake and energy expenditure (Roh et al., 2016). As the primary site for nutrient metabolism and transport, the gastrointestinal tract not only provides essential precursors for MASLD development but also secretes signaling molecules that influence interactions among hepatic and extrahepatic organs (Ezquerro et al., 2018). Meanwhile, the pancreas secretes various hormones and peptides that affect both hepatic and extrahepatic MASLD progression. The skeleton functions as an endocrine organ, with bone-derived factors from osteoblasts regulating hepatic glucose and lipid metabolism via the bone–liver axis (Li et al., 2023b). Skeletal muscle, a major organ for energy expenditure and thermogenesis, is closely associated with metabolic disorders. Given the complexity of this systemic disease, it is essential to investigate these interconnections from an epigenetic (DNA methylation) perspective.

## 7 Conclusion and perspectives

Evidence from human and animal studies clearly indicates that the onset of MASLD is influenced by genetic and epigenetic factors. In this review, we closely examine genetic variants in lipid metabolism-related genes that are significantly associated with MASLD and MASH. Additionally, we explore relevant animal models and the alterations in DNA methylation of lipid metabolism-related genes observed in liver tissue and peripheral blood leukocytes of MASLD and MASH patients. It is important to recognize that genetic and epigenetic factors should not be viewed as two independent categories that operate without interaction. Existing study has identified a close relationship between DNA methylation and nucleotide variation. For instance, the genetic variant at the rs738409 locus in the PNPLA3 gene is strongly associated with methylation changes at CpG_99, while the genetic variant at the rs174616 locus in the FADS2 gene is closely related to methylation alterations at cg07999042 (Kitamoto et al., 2015; Walle et al., 2019). Interestingly, in our comprehensive analysis of SNPs and DNA methylation information related to lipid metabolism genes significantly associated with MASLD and MASH, we found that both genetic variants and differential DNA methylation cites were mapped to ACSL4, APOB, PPARα, PPARγ, and SLC27A5 genes, which play a crucial role in the progression of MASLD and MASH. Additionally, we briefly reviewed the relationship between genetic variants and DNA methylation of lipid metabolism–related genes and immune response, as well as the interplay between extrahepatic signals from other tissues and DNA methylation of lipid metabolism–related genes in MASLD and MASH.

It is noteworthy that non-invasive detection methods hold considerable value in clinical practice. Compared to liver biopsy, blood tests are more readily accepted by patients (Powell et al., 2021). The genomic DNA derived from leukocytes and cfDNA in peripheral blood contain extensive genetic and epigenetic (DNA methylation) information. The ability to identify valuable biomarkers from this information for stratifying MASLD will aid in its diagnosis, prognosis, and therapeutic interventions. Currently, numerous studies predominantly employ bisulfite conversion-based sequencing methods to investigate DNA methylation modifications in liver diseases. However, these methods present unavoidable limitations, primarily associated with the handling of bisulfite (Tanaka and Okamoto, 2007). Firstly, over half of the DNA molecules may degrade during bisulfite treatment, restricting their applicability in rare and precious samples. Secondly, the conversion of unmethylated cytosines (which account for approximately 95% of the human genome) to uracils can adversely affect the base composition of DNA sequences, leading to reduced accuracy in sequencing data and diminished effectiveness in genomic alignments. Additionally, samples subjected to bisulfite treatment are often unsuitable for detecting SNVs. Consequently, the same sample must undergo two distinct assays to evaluate SNVs and DNA methylation levels, complicating the investigation of the relationships between genetic and epigenetic factors.

A technology that can conveniently and accurately detect SNP and DNA methylation information in the blood of large clinical cohorts will greatly enhance our understanding and exploration of the intricate relationships between genetic and epigenetic factors affecting diseases. Fortunately, with the ongoing advancements in sequencing technology, third-generation long-read sequencing has been introduced in epigenetic research. Notably, Oxford Nanopore Technologies (ONT) has developed a third-generation sequencing technique known as nanopore sequencing (Simpson et al., 2017). This technique’s core involves guiding DNA molecules into the nanopore channels on a sequencing chip (flow cell). As these molecules pass through the nanopores, different bases and methylation states generate characteristic current signals, which can be measured to simultaneously and accurately obtain information on SNP and methylation states. This technology has gained widespread application in recent years due to its advantages of single-molecule real-time sequencing and ultra-long read lengths (Zhang et al., 2021). In DNA methylation detection, this technology exhibits numerous advantages over conventional methods regarding read length, throughput, speed, and the requirement for DNA sample pre-treatment (such as bisulfite conversion and PCR amplification) in detecting 5 mC modifications. Consequently, nanopore sequencing is positioned as an ideal technology for the concurrent detection of gene variants and DNA methylation status. Furthermore, in the context of large patient cohorts, specific barcodes can be ligated to different patient samples during the library preparation process, allowing sequencing on the same sequencing chip (flow cell). Subsequently, these samples can be distinguished during data analysis. To improve the sequencing depth of targeted regions of interest and reduce sequencing costs, ONT has introduced their Cas9 protocol for targeted sequencing, utilizing CRISPR-Cas9 technology to selectively cleave and enrich specific gene regions for sequencing. Several studies have successfully employed this technology, achieving desired information and significant outcomes (Gabrieli et al., 2018; Gilpatrick et al., 2020; Stangl et al., 2020; Wongsurawat et al., 2020; Stevanovski et al., 2022; Wieting et al., 2023). It is worth noting that with the development of bioinformatics technologies, analysis software based on nanopore long-read sequencing and nCATS sequencing data has been gradually developed, including Nanopolish, f5c, DeepSignal, DeepMP, DeepMod, Tombo, Megalodon, Dorado, and DeepMod2, as described in Ahsan et al. (2024), which provide powerful tools for identifying methylation sites of target genes. Therefore, the simultaneous detection of DNA methylation and genetic variants information in peripheral blood through nanopore sequencing technology is anticipated to become an important clinical tool in the future.

Overall, these efforts aim to provide vital insights into how genetic variants and DNA methylation influence the genes involved in lipid metabolism, leading to the onset and progression of MASLD and MASH. Furthermore, the pursuit of accurate and sensitive biomarkers for the stratification of MASLD will significantly aid in the diagnosis, prognosis, and therapeutic interventions for MASLD, thereby promoting the advancement of personalized medicine.
